# Drug Delivery Device Design and Compatibility with Nitrogen Dioxide Gas Sterilization

**DOI:** 10.3390/ph18121869

**Published:** 2025-12-08

**Authors:** Noelle Ray, Julia Diane Schexnayder, Aiysha Ashfaq, Nusrat Sarwahrdy, Delaney Lisco, Minufar Abdollahi Khabisi, Trevor Bateman, Tom Sadler, David Opie, Mohamad Al-Sheikhly

**Affiliations:** 1 Noxilizer, Inc., 1334 Ashton Road, Suite E, Hanover, MD 21076, USAdlisco@noxilizer.com (D.L.); mabdollahi@noxilizer.com (M.A.K.);; 2 Department of Materials Science and Engineering, University of Maryland, College Park, MD 20742, USA; schexnj@umd.edu (J.D.S.); trevorbateman.school@gmail.com (T.B.)

**Keywords:** sterilization, polymers, autoinjector, medical device, prefilled syringe, drug delivery device, terminal sterilization, gas sterilization

## Abstract

Polymeric materials have become important components in prefilled syringes, drug delivery systems, and advanced medical devices. **Background/Objectives**: Nitrogen dioxide gas is used for the terminal sterilization of drug delivery systems. For the implementation of sterilization methods, compatibility with materials must be demonstrated such that the materials maintain product requirements and specifications after sterilization and at the time of use (i.e., product shelf life). **Methods**: Commonly used polymers were selected based on their chemical structures to provide insight into the nature of reactions that occur at the temperature and NO_2_ concentration levels used in the sterilization process. After exposure to the NO_2_ process, materials were evaluated for chemical, mechanical, and biocompatibility properties. **Results**: In this paper, we demonstrated the compatibility of polymers comprising carbonyl, unsaturated ester, and ketone groups which have been used in medical devices sterilized with NO_2_. No significant chemical or physical changes were observed upon the treatment of Amorphous Polyester, Polysulfone (PSU), Polycarbonate (PC), PolyEtherEtherKetone (PEEK), PolyArylEtherKetone (PAEK), and Polypropylene (PP) with NO_2_ at a sterilization temperature of 20 °C. At this relatively low sterilization temperature, the reactions of NO_2_ with the polymer do not typically occur because the activation energies of these reactions require much higher temperatures. **Conclusions**: Not all materials will be compatible with NO_2_ sterilization, and even with the established data, many devices will need to have their polymers evaluated for compatibility before moving to NO_2_ sterilization. These results will provide guidance to device designers selecting materials for new drug delivery devices and to regulators that review the safety and efficacy of these devices.

## 1. Introduction

Biologic therapeutics, including monoclonal antibodies, recombinant proteins, and cell- or gene-based therapeutics, are often delivered via drug delivery devices such as prefilled syringes and autoinjectors [1]. To meet the requirements for patient safety, some drug delivery devices require terminal sterilization after aseptic filling [2]. This terminal sterilization targets the surfaces of the drug delivery device and is not a sterilization of the biologic contained within. The need for terminal sterilization comes from the criticality of the application. For example, prefilled syringes used for ophthalmic injections (intravitreal injections) are terminally sterilized after filling and packaging to render the external surfaces of the syringes sterile to reduce the risk of intravitreal infection [3]. Additionally, autoinjectors may undergo terminal sterilization where microbial contamination in the fluid path would pose a risk to immunocompromised patients.

The sterilization method used for the drug delivery devices must be selected with consideration for the therapeutic agent in the filled drug delivery device and for the sterilization compatibility of the materials used in the construction and packaging of the device. Candidate sterilization methods include radiation sterilization (gamma, X-ray, or e-beam) and gas sterilization, including ethylene oxide (EO), hydrogen peroxide (H_2_O_2_), and nitrogen dioxide (NO_2_). There are many papers regarding the radiation sterilization of polymers but very few about gas sterilization’s compatibility with polymers [4,5,6]. However, terminal sterilization using ionizing radiation will degrade pharmaceutical and biologic agents contained in the drug delivery devices and is typically not suitable for terminal sterilization [4]. Therefore, there is a need for detailed analyses of the nature of chemical reactions observed with the gas sterilization of polymers [4,5,6].

Some gas sterilization methods can cause degradation of the therapeutic agent within the drug delivery device. The mechanisms of degradation with gas sterilization may result from the elevated temperature of the sterilization process and from the gas sterilant passing through the primary container closure and contaminating the biologics within [7].

Regarding the temperature degradation of thermolabile biologics, the bioactivity of biologics can be reduced by elevated temperatures during processing, storage, and transit, as aggregation, denaturation, and oxidative degradation render therapeutic molecules ineffective [8]. EO sterilization will use a process temperature that is greater than 40 °C and H_2_O_2_ sterilization will require process temperatures greater than 30 °C, whereas NO_2_ sterilization has been validated at as low as 18 °C. The potential degradation caused by elevated temperature sterilization processes will follow the Arrhenius equation, meaning that increasing process temperature will increase the degree of degradation [9]. Therefore, the relatively low process temperature of the NO_2_ sterilization process will be less degradative for biologics.

The degradation of the biologics caused by contamination with the sterilant gas entering the primary container has been found to occur with both EO and H_2_O_2_ sterilization [7]. Conversely, the NO_2_ sterilization process did not result in this degradation of proteins. The reason for this is that NO_2_ does not pass through the bulk of polymeric materials like radiation nor permeate through materials as easily as EO [10,11]. The permeability of hydrogen peroxide through materials has also been reported, which should be taken into consideration as a potential mode for degradation of the biologics [12].

In addition to permeability, the magnitude of the pressure fluctuations during the EO and H_2_O_2_ sterilization processes span from ambient pressure to less than 100 mbar and as low as 1 mbar for H_2_O_2_, causing the piston in a prefilled syringe to move, which may contribute to contamination of the syringe contents and degradation of the proteins in the reported studies [13,14]. Given the lower process temperature and ability to use a process with a small magnitude of pressure fluctuations, NO_2_ sterilization has gained acceptance as a gas sterilization method for drug delivery device terminal sterilization [15].

Another consideration for the design of drug delivery devices is the material compatibility of the drug delivery device with the intended terminal sterilization method. Prior published data on the chemical reactions observed with polymeric materials exposed to NO_2_ considered the exposure of such polymeric materials to atmospheric pollutants, including NO_2_ [16]. The concentration of NO_2_ in the environment is low (less than 1 ppm), and as such, the rates of observed reactions with polymers are slow. To accelerate the nature of potential interactions between polymers and atmospheric levels of NO_2_, the published data reported on reactions observed using elevated temperatures and elevated NO_2_ concentration levels so that the reactions are amplified compared to true atmospheric exposure [16]. The reported results from these studies are instructive but do not portray the conditions that polymeric materials will encounter during an NO_2_ terminal sterilization process. The objective of this report is to share data on polymer compatibility with NO_2_ sterilization and to describe the types of chemical reactions that can occur when polymers are exposed to NO_2_ at the sterilant-relevant temperature and concentration levels. An understanding of these reactions will further facilitate the design of drug delivery devices that use NO_2_ for terminal sterilization.

### 1.1. NO_2_ Sterilization Process

NO_2_ is a gaseous sterilant which, when combined with humid air, can sterilize medical devices [17]. The NO_2_ process has been validated, and marketed drug delivery devices use the NO_2_ terminal sterilization method [18]. In support of the validation, the biological indicators used for the NO_2_ sterilization process are shown to exhibit predictable inactivation kinetics, permitting the demonstration of the necessary sterility assurance level (SAL) [19].

**Figure 1:**
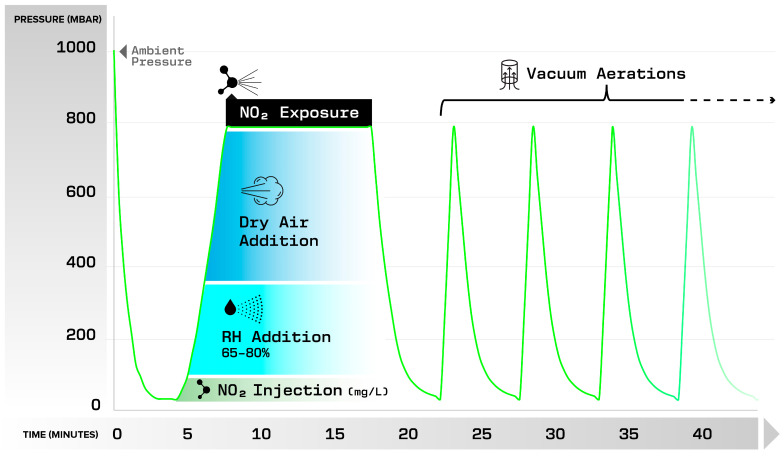
Graph of pressure (mbar) vs. time (minutes) for an example NO_2_ sterilization cycle.

The NO_2_ sterilization process is completed in a vacuum chamber and begins with evacuation of the air in the sterilization chamber. After evacuation, the sterilization chamber is filled with the sterilization process gases which consist of NO_2_ and humidified air. For most medical devices and drug delivery devices that can tolerate a deep vacuum, the chamber is evacuated to a relatively low vacuum pressure (e.g., 20 mbar; see Figure 1). Some drug delivery devices, such as prefilled syringes, are susceptible to piston movement during the sterilization cycle, due to the influence of the evacuated pressure, which causes the gases in the prefilled syringe to expand. The movement of the syringe piston may allow for the contamination of the syringe contents with both microorganisms and sterilant gas. To address this issue, a shallow-vacuum cycle has been developed. This type of shallow-vacuum cycle uses a much higher minimum process pressure (e.g., about 500 mbar minimum process pressure) compared to typical minimum process pressure levels used with deep vacuum cycles.

With the NO_2_ process, both deep vacuum and shallow-vacuum cycles have similar process stages. These stages are described below and illustrated in Figure 1. The process stages are:

- Evacuation: The chamber is evacuated down to the designated minimum process pressure, which removes air from the sterilization chamber, permitting the addition of the process gases.
- Humidity Addition: Relative Humidity (RH) is added to the chamber until the desired percentage is reached, typically between 65% RH and 80% RH.
- NO_2_ Injection: the NO_2_ is metered into the sterilization chamber, with a target concentration typically between 5 mg/L and 20 mg/L.
- Pressurization: Dry air is added to the sterilization chamber to reach the target process pressure (dwell pressure), which is typically between 700 mbar and 800 mbar.
- NO_2_ Exposure Stage: The NO_2_ and RH remain in the chamber for the set dwell time of the NO_2_ exposure. The dwell time ranges from 5 min to 30 min.
- Aerations: Aeration consists of repeated stages of evacuating and refilling the chamber with air until the sterilant is removed from the chamber.

Some sterilization cycles define the stages listed above as a half-cycle, whereby the biological indicators are found to be sterile after completing these stages. For a sterilization full-cycle, these stages are repeated to achieve the needed sterility assurance level, or SAL, as described in Annex D of ISO 14937:2009 [20]. Therefore, a sterilization cycle may consist of two sterilant exposure stages.

To test material compatibility, polymers were exposed to either three full-cycles (six exposure stages), referred to as the Mid-Range Exposure or 3× cycle, or six full-cycles (twelve exposure stages), referred to as the High-Range Exposure or 6× cycle.

### 1.2. NO_2_ Reactions

Chemical reactions between polymers and NO_2_ can be anticipated by examining the polymeric molecular structure. NO_2_ is an oxidizer and a free radical because of the lone electron on the nitrogen atom, written as NO_2_^•^. At the temperature and concentration of the NO_2_ sterilization process, the possible oxidative reaction mechanisms with polymers include addition to the double bond pi structure of a phenyl group, the abstraction of hydrogen from the backbone of the polymeric chain, and electron transfer mechanisms [21].

For example, with nylon and polyurethane, the N–H group in the polymer chain is susceptible to oxidation by NO_2_ because of the functional amide group in nylon (–CO–NH–) and the carbamate group in polyurethane (–NH–CO=O–) [22]. In Delrin (polyoxymethylene, or POM), the oxidative degradation pathways lead to chain scission at the methylene group (–O–CH_2_–) and result in formaldehyde release [23].

**Figure 2:**
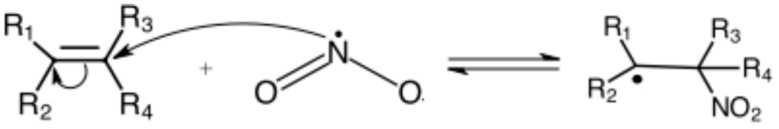
NO_2_ addition reaction.

As an example, NO_2_ can add to double-carbon bonds in a polymer backbone, leading to the formation of a c-centered radical (Figure 2).

**Figure 3:**
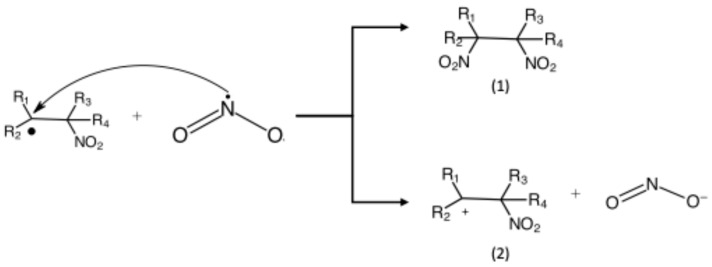
(1) NO_2_ addition reaction or (2) Electron Transfer Mechanism.

The resulting reducing C-centered radical may undergo further oxidizing reaction to produce the cation and NO_2_- ions or produce di-nitro compounds and nitro nitriles (Figure 3). In this example, the reaction between the double-carbon bond and the NO_2_ resulted in radical formation. 

As a further example of potential reactions, NO_2_ can react with the vinylene and vinylidene groups found in polyethylene and polypropylene, respectively. Published studies have also demonstrated that NO_2_ can interact with the phenyl, carbonyl, ester, and ketone groups in various polymers [16].

As a final example of potential reactions, at relatively high temperatures (above 310 K), the reactivity between NO_2_ and polymers increases. It has been reported that carbonyl groups and hydroxyl groups can be formed [24].

Other reactions that might be anticipated are low-level surface reactions, such as carbamate oxidation, acetal cleavage, or amine nitrosation, which may occur when exposed to gaseous NO_2_ under ambient conditions [24]. For polymers with more oxidizable linkages, the overall extent of the reaction is kinetically limited, which is consistent with the observed preservation of mechanical and optical properties following NO_2_ sterilization. For these other reactions, and despite the oxidizing properties of NO_2_, reactions between NO_2_ and these polymers require overcoming activation-energy barriers, which can be achieved only at elevated temperatures [16,24].

With this knowledge of possible reactions, we have chosen polymers for this study with carbon backbones, methylene groups, phenyl groups, double-bonded oxygens, and other features that are representative of potential reaction sites with NO_2_. These materials are not reflective of all medical device materials but represent the specific molecular structures we are investigating.

The selected polymers are Amorphous Polyester, Polysulfone (PSU), Polycarbonate (PC), PolyEtherEtherKetone (PEEK), PolyArylEtherKetone (PAEK), and Polypropylene (PP), as representatives of hydrocarbon aliphatic polymers, polycarbonate, and polyester groups. Table 1 summarizes the identifiable molecular structures and functional groups of each material, along with the common medical devices in which these materials are used.

This work addresses gaps in the literature regarding NO_2_ gas exposure at lower temperatures (10–30 °C), high concentrations, and long exposure times to extensively evaluate the materials and their compatibility with the NO_2_ sterilization process. This work is only the start of answering gaps in the literature. Additional polymers will be considered elsewhere and will include studies of exposed polymers aged in real time (not accelerated).

## 2. Results

### 2.1. NO_2_ Achieved Parameters

All material exposure cycles were completed according to the parameters and tolerances listed in table of Section 4.4. A total of seven Mid-Range exposure cycles and ten High-Range exposure cycles were completed. The average exposure conditions achieved are summarized in Table 2. The vacuum level reached 20 torr for all cycles. The exposure pressure was consistently 592 torr, which is 2 torr above the targeted pressure but is within the set tolerance. The relative humidity was also consistently at 79.6%, which is only 0.4% off the target. While most validated cycles have a temperature range 18–22 °C, the material exposure cycles in this study had an average exposure temperature of 24 °C, providing a “worst case” for polymer exposure.

The uncertainty for all cycle parameter values was calculated from the standard deviation of all the NO_2_ concentrations achieved for each group.

### 2.2. NO_2_-Induced Chemical Reactions

#### 2.2.1. Surface Characterization Changes

**Figure 4:**
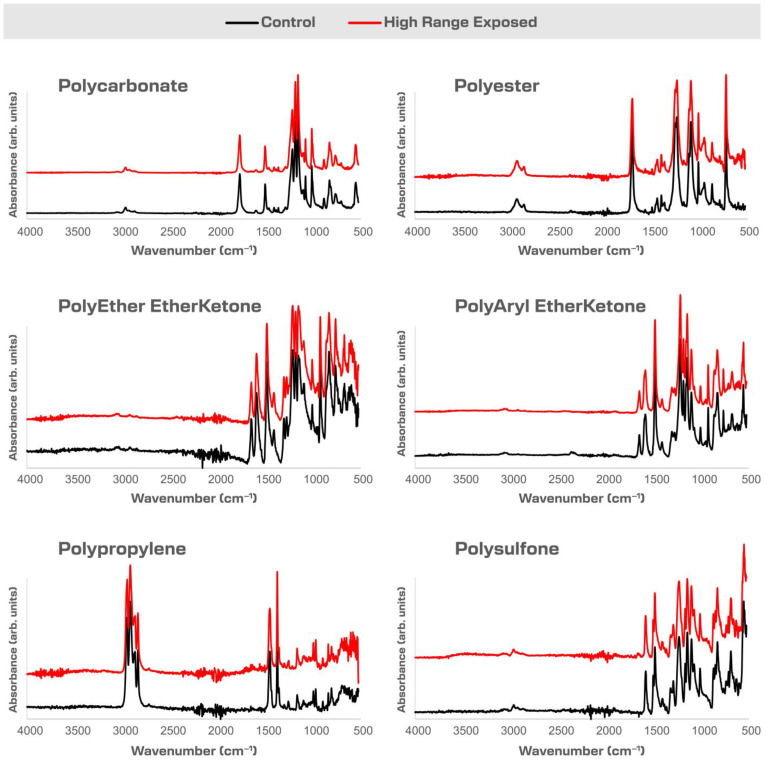
FTIR summary spectra for each polymer sample control, High-Range-exposed.

The surface of each material was characterized using FTIR-ATR Spectroscopy, as shown in Figure 4. No significant changes in the FTIR-ATR spectra were observed in the comparison of High-Range-exposed samples with control samples. The variations were analyzed using a two-sample *t*-test using a 95% confidence interval, which showed no shift of absorbance wavenumbers or significant changes in absorbance (peak height). Insignificant variations in peak height (less than 5%) are attributed to instrumental factors such as spectral resolution, signal-to-noise ratio, and variability in ATR contact rather than actual differences in the samples themselves [37,38]. This indicates that there were no molecular changes in the polymers tested after NO_2_ exposure.

#### 2.2.2. Free Radicals

**Figure 5:**
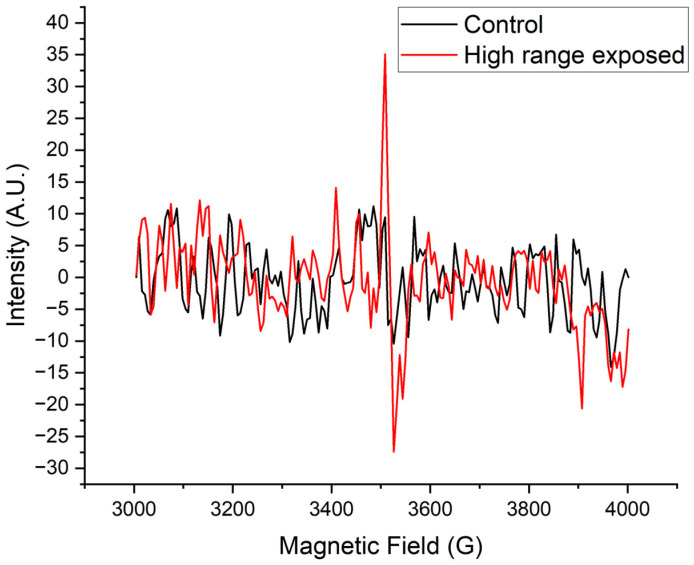
EPR spectra of Amorphous Polyester before NO_2_ exposure (red) and after NO_2_ exposure (black). The exposed sample exhibited an EPR spectrum with a g-value of 2.0029.

Each material was analyzed for the presence of free radicals using EPR. Only the NO_2_ High-Range-exposed Amorphous Polyester samples exhibited a signal at 3515 G, as shown in Figure 5. For this signal, the g-value was calculated using Equation (3) and was found to be 2.0029. The number of radical electron spins per gram of material was calculated with Equation (4) and was found to be 1.71 × 10^14^ spins/g.

#### 2.2.3. Change in Morphology

The morphology of each material was evaluated using DSC. For amorphous materials, the glass transition temperature (T_g_) was measured, and for semi-crystalline materials, the melting temperature (T_m_) was measured. All reported peaks agree with previously reported literature values. The variations were analyzed using a two sample *t*-test using a 95% confidence interval, which showed that no samples exhibited a significant difference in melting or glass transition temperatures (as is shown in Table 3 and Table 4), indicating no change in bulk morphology with NO_2_ exposure. The reported literature values for the glass transition temperature for Polyester [39], PSU [40], and PC [41] are found in Table 3, and the reported literature values for the melt temperature of PEEK [42], PAEK [43], and PP [44] are found in Table 4.

For the semi-crystalline polymers, the crystallinity of the test samples was calculated using literature reference values for 100% crystalline Polypropylene [45], PAEK [46], and PEEK [47]. There was no significant difference between the percent crystallinity of Mid-Range-exposed samples and High-Range-exposed samples when compared to the control percent crystallinity (Table 5), further confirming the lack of change in the polymer structure upon NO_2_ exposure.

#### 2.2.4. Mechanical Changes

Changes in the mechanical properties of exposed materials were evaluated using measured mechanical properties from tensile testing. For the five samples tested from each group, the average true yield strength, true strength at fracture, and true percent elongation at fracture of each polymer are compared in Table 6. Elastic Modulus and strain at yield or fracture are not reported since an extensometer was not used during testing. A comparison of the Mid-Range-exposed samples and High-Range-exposed samples to the control samples shows no significant difference (significance level = 0.05) using an unpaired two-tailed *t*-test.

#### 2.2.5. Shore Hardness

No significant change in hardness (Durometer) was observed between the control, Mid-Range-exposed, and High-Range-exposed samples. The Shore D hardness values for the materials Amorphous Polyester, PSU, PC, PEEK, PAEK, and PP are summarized in Table 7.

#### 2.2.6. Color Change

The materials in this study were evaluated for visual change using a color reader that provided a ΔE value, comparing the color of the control samples with the exposed samples. The results from these measurements are shown in Table 8. Polypropylene and Polycarbonate exhibited the greatest degree of color change, with a ΔE value greater than 4. The other samples had a ΔE value less than 2 after exposure.

#### 2.2.7. Surface Residuals

All materials were extracted in the specified manner (see Materials and Methods, Section 4.5) and the extraction medium was evaluated for the concentration of extracted nitrates and nitrites. The limit of quantification (LOQ) was 0.043 ppm for nitrates and 0.038 ppm for nitrites, translating to 0.034 µg/cm^2^ and 0.030 µg/cm^2^, respectively. The values of extracted sterilant residuals are shown in Table 9. All the controls were low, less than 0.660 ug/cm^2^ for nitrates and 0.0746 ug/cm^2^ for nitrites.

The nitrates are average values of the 6×exposed group and the nitrite values are the averages of 3× and 6× groups based on the highest average measured for each group.

#### 2.2.8. Biocompatibility

The observed cytotoxicity grade, based on the resulting cell culture conditions corresponding to the numerical cytotoxicity grade for each material, can be found in Table 10. The interpretation of the grade is in table of Section 4.5. All other materials were found to be non-cytotoxic.

## 3. Discussion

### 3.1. FTIR

**Figure 6:**
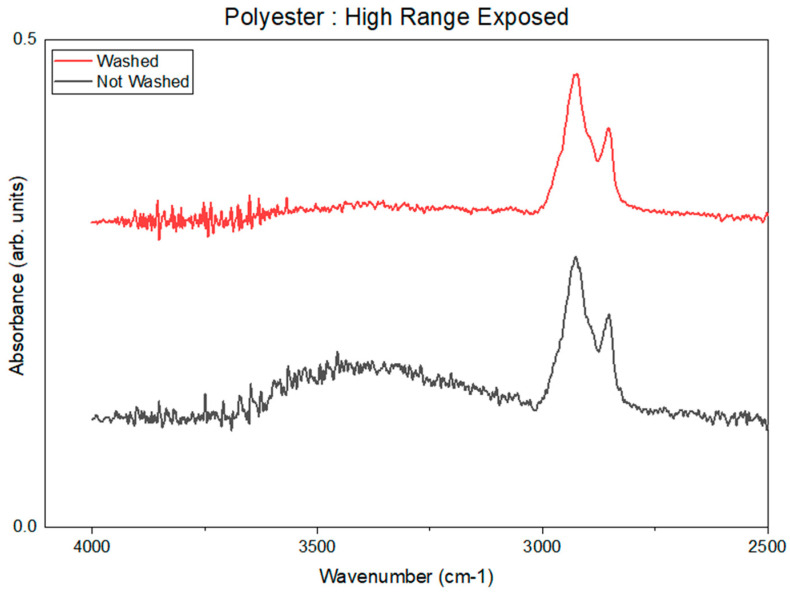
Example of signal around 3500 cm^−1^ for the High-Range-exposed sample washed and not washed for Amorphous Polyester.

The FTIR-ATR measurements did not detect chemical changes in the polymers. However, an absorbance band in the range of 3200 cm^−1^ to 3750 cm^−1^ was observed. This absorbance band is shown in Figure 6 and suggests the presence of O-H or N-H stretching vibrations. However, following the washing of samples with water, this absorbance band was not detected. This observation correlates with the analysis of the sterilant residuals, extracted in aqueous solutions. Because we can extract varying levels of nitrate and nitrite ions, as shown in Table 9, we know there is residual nitrate and nitrite on the polymer surfaces. This suggests that the observation of absorbance in the range of 3200 cm^−1^ to 3750 cm^−1^ after exposure is not a result of a chemical reaction; rather, sterilant residue was present on the material surfaces.

Polyethylene (PE), Amorphous Polyester, PAEK, and PEEK each show a change in the spectrum from 3200 cm^−1^ to 3750 cm^−1^. PC did not show a change in spectra between unexposed and exposed samples.

The NO_2_ sterilization process uses an atmosphere of NO_2_ and humidified air. It is known that humidity interacts with materials, forming monolayers of water, within which the reactions between water and NO_2_ occur [17]. The thickness of the water monolayers on a material surface is dependent on the surface material and the partial pressure of water at the surface [48]. The monolayers of water form because the process adds humidity to the chamber, resulting in water molecules forming these monolayers on the material surfaces. When NO_2_ gas encounters water, one of the reactions that can occur is the formation of two acids: HNO_3_ (nitric acid) and HNO_2_ (nitrous acid), as shown in Equation (1), below.

2NO_2_(g) + H_2_O(aq) → HNO_3_(aq) + HNO_2_(aq) (1)

HNO_2_(aq) → NO(g) + HNO_3_(aq) + H_2_O(l) (2)

HNO_2_ is inherently unstable and decomposes rapidly into NO (nitric oxide), HNO_3_, and additional water (Equation (2)). The instability of HNO_2_ is illustrated in the sterilant residual testing results shown in Table 9. In this table, the amount of recovered nitrite is much lower than that of nitrate, regardless of the expectation of equal stoichiometry implied by Equation (1).

At the end of the sterilization process, the vacuum in the sterilization chamber vaporizes water, leaving an amount of HNO_3_. The HNO_3_ contains a highly polar –OH group and a strongly electronegative –NO_2_ moiety. On polymers with hydrogen bond acceptors (e.g., –C=O, –O–, –N–), HNO_3_ can form directional hydrogen bonds. When immersed in water, the water can break these hydrogen bonds through competitive solvation and molecular displacement. Therefore, this is not a chemical reaction between NO_2_ and the polymers.

### 3.2. EPR

**Figure 7:**
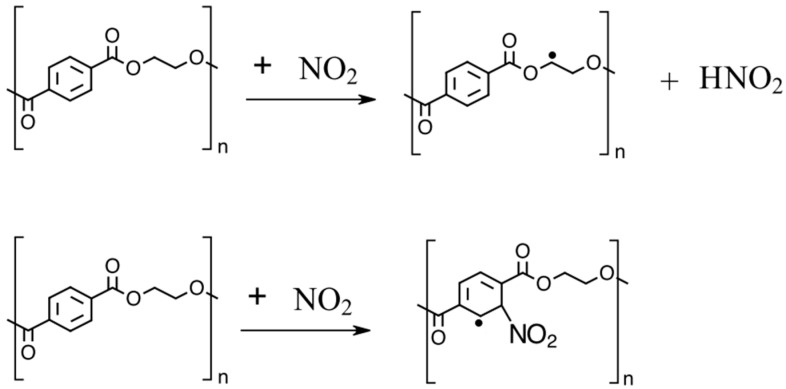
Possible reaction mechanisms between NO_2_ and polyester.

The EPR spectrum shown in Figure 5 displays a signal with a g-value of 2.0029, which is consistent with carbon-centered radicals, particularly those adjacent to electronegative atoms such as oxygen [49,50]. Because there is no oxygen neighboring a carbon in the phenyl group, we conclude that the NO_2_ abstracts a hydrogen atom from the polyester backbone and not the phenyl group. The potential reactions between NO_2_ and the Polyester backbone are shown in Figure 7.

This observation of an EPR signal is important because it could elucidate a critical polymer degradation pathway. The published literature on this topic has numerous examples of degradation mechanisms leading to decreased tensile strength, embrittlement, and reduced molecular weight, the effects of which may not be immediately apparent but manifest as long-term aging and performance loss. This is caused by the free radicals generated in polymers during radiation sterilization. In the presence of oxygen, these primary C-centered free radicals react to form peroxyl radicals, which then undergo further reactions, leading to the degradation of the polymer chains, particularly at low dose rates common in some sterilization processes [51].

After the first sample of Amorphous Polyester was observed to exhibit radicals in the EPR spectrum, subsequent testing was performed. This included three (3) samples of Polyethylene Terephthalate Glycol (a modified Polyester), three (3) samples of Crystalline Polyester, and two (2) samples of Amorphous Polyester. All samples in this subsequent testing were placed on dry ice immediately following NO_2_ exposure until EPR testing. Only one of the two samples of Amorphous Polyester during this subsequent testing exhibited an EPR signal showing radical formation.

The two samples of Amorphous Polyester that had shown evidence of radicals were retested: the first retest after 25 days and the second retest after 6 days. No EPR spectrum was observed in either sample, suggesting that the C-centered radicals undergo crosslinking or some other relaxation reaction and decay rapidly.

No changes in functional properties were observed for the Amorphous Polyester, and the material only exhibited low surface residual levels and a non-cytotoxic response. Other Crystalline Polyester samples from different batches did not exhibit EPR signals. The lack of EPR signal may be because the Crystalline Polyester samples contain certain antioxidants, or the NO_2_ diffusion through the sample bulk was impeded because of the crystallinity. Table 11 lists the additional EPR testing performed and the material information of the samples tested.

### 3.3. Tensile Testing and Shore Hardness Durometer

There was no significant change in mechanical properties or in the measured hardness after NO_2_ exposure with the materials reported herein. The value of establishing a correlation would be for screening material impact with a simple hardness test. Other published reports have correlated changes in hardness to changes in mechanical properties. It has been shown that changes in polymer durometer can occur from cross-linking after radiation sterilization [5]. Additionally, research from Tomas Bata University examined polyamide 12 (PA12) irradiated with e-beam, where they found that nano-hardness increased by 61% at 132 kGy, directly correlating with increased crosslink density [5]. A white paper from Saint-Gobain Performance Plastics studied the effects of EO sterilization on silicone rubbers. While EtO is less aggressive than radiation, the paper notes that crosslinking reactions can still occur, especially in peroxide-cured silicones, resulting in increased durometer hardness and tensile modulus [52]. However, for the materials reported here, we did not observe changes in mechanical properties or in hardness. Therefore, the correlation between mechanical properties and hardness is inconclusive.

Across all polymers, changes are within ±1 Shore D unit, which is well within measurement variability. This suggests that NO_2_ sterilization does not cause substantial embrittlement or softening, preserving mechanical properties. Hardness correlates with surface durability and resistance to deformation. Since hardness remains stable, the polymers should maintain their load-bearing capability, wear resistance, and dimensional stability post-sterilization.

### 3.4. Color Change

The materials in this study were evaluated for visual change using a color reader that provided a ΔE value by comparing the difference between control and exposed samples. ΔE (Delta E) is a value that quantifies the perceptual difference between two colors. The threshold for what is considered a noticeable color change depends on the ΔE value and the context of viewing. This is described in Table 12 [53]. Only Polycarbonate and Polypropylene had moderate color change after the High-Range Exposure, the rest of the materials had none or only slight color change.

**Figure 8:**
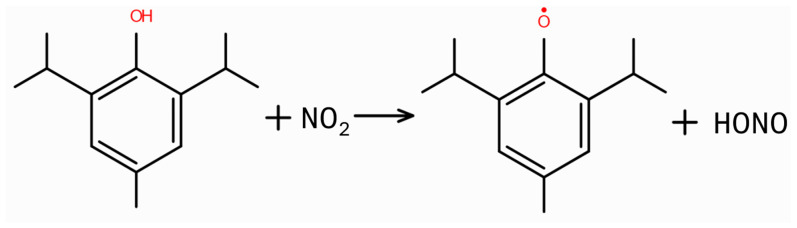
The reaction between the phenolic antioxidant and NO_2_ to make the phenolic hydroxyl group and HONO.

Material color change is observed with other modes of sterilization, most notably following radiation sterilization. To compensate for small color changes, some polymers are blended with the colorants formulated to account for the color change [6]. Color change can be an indication of a reaction of the sterilant with the polymer, or it can be a reaction with the colorants and other additives added to the polymer (e.g., antioxidants, stabilizers, lubricants, etc.) [7]. For example, a common type of organic antioxidant found in medical-grade polymers is phenolic antioxidants [55]. These antioxidants are widely used primary antioxidants in medical-grade polyolefins (like polyethylene and polypropylene) due to their high efficiency, low volatility, and good toxicological clearance [56]. The phenolic antioxidant acts as a free radical scavenger, interrupting the polymer degradation process by undergoing a hydrogen-transfer mechanism. Figure 8 shows the reaction between the phenolic antioxidant and NO_2_.

The resulting alkoxyl radical is less reactive than the original radical and can react further with another NO_2_ radical or undergo other reactions, often leading to nitrated phenols or quinone methide structures, which may cause discoloration (yellowing) of the polymer [57]. This discoloration is not necessarily a sign of polymer degradation but rather the sacrificial transformation of antioxidants. Any additional oxidation removes the unpaired electron from the oxygen, requiring the double-bonding of the O to the aromatic group, resulting in the quinone structure. 

### 3.5. Residuals and Cytotoxicity

Cytotoxicity is a foundational test in demonstrating the biocompatibility of a material [58]. Following NO_2_ sterilization, some level of residuals will remain on the material surface. Residual retention is material-dependent. However, we can use the measured values matched with biocompatibility data to determine levels of residuals that would be harmful. The highest level of residuals shown in Table 9 is for Amorphous Polyester, which had extracted nitrate at 90 µg/cm^2^. This level of residuals was not cytotoxic.

Unlike with EO, there is no established limit for the acceptable level of residuals on a medical device. While none of the NO_2_-exposed materials exhibited cytotoxicity, H_2_O_2_ is found to leave cytotoxic residual chemicals on the surface of polymers. In the report by Ikarashi et al. [59], H_2_O_2_ residuals recovered above 1 µg/cm^2^ were cytotoxic. Similarly, EO residuals are found to be cytotoxic at low residual levels. From ISO 10993-7, the maximum permissible limit of EO residuals is 10 µg/cm^2^ [60]. This is supported by the report from Lucas et al. [61], where they found that materials with about 1 µg/cm^2^ cause a cytotoxic response in cell cultures. These results demonstrate that the sterilant residuals recovered from polymer surfaces after NO_2_ sterilization are less cytotoxic than the same mass density of residuals from either H_2_O_2_ or EO [59].

### 3.6. Material Resistance

Whereas reactions potentially might occur between NO_2_ and the identified molecular structures (e.g., double oxygen bonds, etc.), we did not always observe these reactions. Therefore, we conclude that some materials do not degrade in the presence of an oxidative gas like nitrogen dioxide due to factors like availability and steric hindrance. For example, where one might expect a reaction between NO_2_ and ether linkages (-O-Ar) in Polysulfone, it instead exhibits notable chemical resistance during NO_2_ sterilization, in part due to steric hindrance arising from its rigid, aromatic backbone [62]. The polymer’s repeating unit—composed of phenyl rings flanking sulfone and ether linkages—creates a spatially crowded environment that limits NO_2_ access to reactive sites. This steric shielding reduces the likelihood of hydrogen abstraction and electrophilic attack, which are common oxidative pathways for NO_2_ degradation in polymers such as nylon and polyurethane. Unlike aliphatic or polar polymers, Polysulfone lacks readily accessible hydrogen atoms adjacent to electron-rich centers, and its sulfone group is already in a fully oxidized state, making further oxidation energetically unfavorable. As a result, NO_2_-induced chain scission is minimal. Mechanistically, NO_2_ reactions typically proceed via radical initiation, where NO_2_ abstracts a hydrogen atom to form a polymer-centered radical and HNO_2_.

This paper evaluates compatibility between NO_2_ with selected polymers soon after the polymers were exposed to NO_2_. At the time that this manuscript is being prepared, samples exposed during this study and stored at ambient indoor temperature and humidity are being prepared for testing after one year of real-time aging. Also, additional polymers and elastomers are being evaluated to expand on the learning gained during this testing reported.

## 4. Materials and Methods

### 4.1. Materials

The materials reported herein were selected for their constituent chemical bonds and as representative examples from common polymer groups that are used in medical devices. These polymers are listed in Table 13. As discussed above, these polymers could potentially react with NO_2_ [21,24]. While in no way exhaustive, this group of polymers will provide insight into the general compatibility of polymers with the NO_2_ sterilization process and instruct material selection when designing drug delivery devices.

### 4.2. Material Characterization Methods

The testing of the materials post-exposure to NO_2_ consisted of seven characterization methods. These characterization methods evaluated the change in chemical and mechanical properties after exposure to the NO_2_ sterilization process. These characterization methods were as follows:

1. FTIR–ATR: Fourier Transform InfraRed Attenuated Total Reflectance (FTIR-ATR) Spectroscopy is a technique used to analyze the molecular structure of materials. The FTIR-ATR absorption spectrum was normalized to provide consistent evaluation across samples and groups. FTIR-ATR measures to a depth of 0.5 × 10^−6^ m to 5 × 10^−6^ m.
2. EPR: Electron Paramagnetic Resonance (EPR) was used for evaluating the formation of radicals on the material after NO_2_ exposure. The presence of an EPR signal indicates free radicals in the polymer. Comparing the spectra from control samples with the spectra of exposed samples can detect the creation of radicals in the polymer due to the exposure to the NO_2_ process.
3. DSC: Differential Scanning Calorimetry (DSC) was used to determine whether a change in morphology occurred—specifically, a change in the glass transition temperature or melt temperature from chain scissions or cross-linking. The critical temperatures were evaluated for each material and reported with the uncertainty values. Variations in these measurements indicate alterations in polymer structure, such as changes in crystallinity, crosslinking, or scissions.
4. Mechanical Properties: Tensile testing was used to determine the yield strength, strength at break, and elongation at break for unexposed control, Mid-Range-exposed, and High-Range-exposed samples. The results indicate whether the sterilization process affected the bulk mechanical properties of the materials.
5. Hardness (Durometer): Shore Hardness Durometer was used to evaluate the bulk surface characteristic of the unexposed control, Mid-Range-exposed samples, and High-Range-exposed samples. Shore D was used to measure all of the materials hardness values. Hardness changes can indicate a change in polymer morphology. The change in polymer hardness from cross-linking after radiation sterilization is well studied [5].
6. Color Change: Color change (ΔE) was measured between the control and Mid-Range-exposed samples and between and the control and High-Range-exposed samples. The exposure of polymers to NO_2_ can lead to discoloration (yellowing). This discoloration has multiple mechanisms, including reaction with additives, oxidative degradation, and surface adsorption [63]. NO_2_ reacts with additives like phenolic-type stabilizers in the polymer matrix, forming quinone-like structures or nitroso derivatives [64]. These compounds absorb light in the visible spectrum, causing a yellow or pink tint. Additionally, NO_2_ is an oxidizer and can initiate chain scission or crosslinking, which can alter the polymer’s chemical structure and create chromophoric groups that absorb visible light. Finally, NO_2_ can permeate into the polymer surface and cause color change from the NO_2_ molecules present in the material.
7. Surface Residuals: Surface residuals were measured using a colorimetric Griess reagent assay. Relating the level of surface residuals (nitrates and nitrites) to the cytotoxicity provides insight into whether biocompatibility will depend on reactive byproducts between NO_2_ and the polymer blend (including additives), or from the sterilant residuals. Published literature has shown that sterilant residuals are cytotoxic above threshold levels [59,61].
8. Cytotoxicity: Cytotoxicity was used as an indicator of biocompatibility, combined with measured surface residuals (nitrates and nitrites). Cytotoxicity was performed in an external laboratory following GLP measures.

### 4.3. Material Preparation

All polymeric materials were received from the material suppliers in the form of ASTM Type 1 tensile bars [65]. The samples were inspected, cleaned by wiping with distilled water, and then air-dried. The dry samples were sealed in Tyvek/Mylar pouches (A7320 Roll Stock from Beacon Converters) and labeled. The packaged samples were divided into three groups. The first group was set aside as unexposed control samples. A second group of samples was exposed to the Mid-Range NO_2_ exposure cycle, while the third group of samples were exposed to the High-Range NO_2_ exposure cycle, following the parameters outlined in Table 2.

Samples for DSC and EPR testing had to be cut from the tensile bars into smaller pieces, weighing less than 5 mg. These samples were wiped with distilled water and air-dried before being placed in pouches and labeled.

### 4.4. Material Exposure

The exposure cycles were completed in the 730 L volume R&D sterilizer (Noxilizer Model RTS-360, Noxilizer, Hanover, MD, USA). The exposure parameters are listed in Table 14.

### 4.5. Post-Exposure Handling and Testing

After the exposure cycles were completed, the samples for FTIR-ATR testing were washed to remove exposure process residues. This washing process consisted of placing the samples in extraction bags with water and incubating for 72 h +/− 2 h at 50 °C, following ISO 10993-12 instructions for material extraction [66]. This process was adopted for sample washing because this is the washing process used to extract residuals from the material surfaces for the residual sterilant testing and for the testing cytotoxicity of the materials. For EPR, used for the detection of radicals like NO_2_, washing the NO_2_ from the surface would remove any signal from NO_2_ persisting within the material, hindering the detection of NO_2_ free radicals that could contribute to ongoing degradation.

After drying the washed samples, the samples were placed in resealable bags and transported to the University of Maryland (UMD) Department of Materials Science and Engineering Laboratory for Radiation and Polymer Science. The FTIR-ATR testing was performed using a Thermo-Scientific Nicolet IS50 FTIR following ASTM E168-16 (2023) [67]. Five control samples, five Mid-Range-exposed samples, and five High-Range-exposed samples were evaluated with FTIR-ATR. The spectra from control samples were compared to the spectra of exposed samples. The changes in the FTIR spectra were reviewed to identify new or missing chemical bonds in the polymer.

The EPR samples were transported to the UMD Laboratory for Radiation and Polymer Science. One control sample and one High-Range-exposed sample were evaluated with EPR. The EPR spectra of all samples were measured using a Bruker EMX EPR spectrometer (Bruker, Billerica, MA, USA), equipped with an X-band (9–10 GHz) microwave source. EPR spectra were measured 24 h after exposure to NO_2_. All measurements were conducted at room temperature. 2,2-diphenyl-1-picrylhydrazyl (DPPH) was used as a standard for the quantification of spins within the polymer samples tested. The g-value for the EPR results was calculated using Equation (3):

(3) $$\begin{array}{c} g=\frac{hv}{\mu_{B}\times B} \end{array}$$

Here, *h* is Planck’s constant, *ν* is the microwave frequency, *μ_B_* is the Bohr magneton, and *B* is the magnetic field strength at which resonance occurs. The number of spins in the sample was calculated by integrating the EPR signal of a polymer sample and comparing this to the standard sample of DPPH with a known spin concentration (Equation (4)).

(4) $$\begin{array}{c} \left[x\right]=\frac{\left[std\right]A_{x}R_{x}{\left(scan_{x}\right)}^{2}G_{std}M_{std}{\left(g_{std}\right)}^{2}{\left[s\left(s+1\right)\right]}_{std}}{A_{std}R_{std}{\left(scan_{std}\right)}^{2}G_{x}M_{x}{\left(g_{x}\right)}^{2}{\left[s\left(s+1\right)\right]}_{x}} \end{array}$$

In Equation (4), [*std*] is the known concentration of the spins/g in the DPPH, A represents the area under the EPR absorption curve, and R is the receiver gain. The term g refers to the g-value of the radical (Equation (3)), which serves as a unique fingerprint for identifying radical species, and s is the electron spin quantum number. The instrumental parameters used in the calculation include G, the modulation frequency (measured in Hz); M, the modulation amplitude (in Gauss); and scan, the magnetic field sweep width (also in Gauss). The subscripts “std” and “x” refer to the standard DPPH sample and the experimental polymer sample, respectively.

Differential Scanning Calorimetry (DSC) using a Perkin Elmer PYRIS Diamond DSC was used to assess the changes in morphology induced by NO_2_ exposure by evaluating changes in melting temperature (T_m_), crystallization temperature (T_c_), or glass transition temperature (T_g_). This characterization followed ASTM D3418-21 [68]. Three control samples, three Mid-Range-exposed samples and three High-Range-exposed samples were transported to the UMD Laboratory for Radiation and Polymer Science to be evaluated using DSC. The DSC testing program consisted of an initial heat ramp above T_m_ to erase thermal history, a cooling ramp back to the starting temperature (below T_g_), and a final heat ramp above T_m_. All heat ramps were run at 10 °C/min. Peaks were identified by analyzing the derivative of the second heat ramp heat flow curve.

Mechanical properties were characterized by measuring yield strength, strength at fracture, and elongation at fracture using the Tinius Olsen H25KT Universal Testing Machine, following guidance from the ASTM D638-22 [65]. Five control samples, five Mid-Range-exposed samples, and five High-Range-exposed samples were transported to The UMD Laboratory for Radiation and Polymer Science to be evaluated for mechanical property characterization. The samples were fixed in the jaws to align with the end tabs. The machine was set to a strain rate of 50 mm/min and recorded force (N) as a function of displaced position (mm). The data were analyzed by graphing the true stress and strain from the force and displacement data. The graph was used to determine the yield strength, strength at break, and elongation at break. Uncertainty using a 95% confidence interval test is reported, and an unpaired two-tailed *t*-test was used to evaluate the consistency of the data [69].

The hardness characterization of materials reported here used a PosiTector Shore Hardness Durometer (Model SHDD1) with a DeFelsko Shore Hardness D Durometer Probe (DeFelsko, Ogdensburg, NY, USA) following the Durometer Hardness Test described in ASTM D2240-15(R21) [70]. For each material, hardness was tested 15 times: five tests on each of the three samples from each of the control, Mid-Range-exposed and High-Range-exposed samples. The DeFelsko Shore Hardness D Durometer probe is a hardened steel rod with a 30° angle conical point indenter. Uncertainty, calculated using a 95% confidence interval test, is reported.

Color change analysis was performed using a Konica Minolta Color Reader CR-10 Plus (Konica Minolta, Tokyo, Japan) with a White Tile and Polaroid Photo Box. The procedure was guided by ASTM E1347-06 (2020) [71]. The measurement of ΔE is a comparison of two scans, which in this study is a comparison of a scan of a control sample to the scan of a Mid-Range- or High-Range-exposed sample. Three samples for Mid-Range-exposed and High-Range-exposed groups were evaluated against a control sample. For each sample, color was tested three times. Uncertainty calculated using a 95% confidence interval test is reported.

Surface Residuals Analysis employed a colorimetric method (Griess Reagent assay) to quantify the extracted residuals, such as nitrate (NO_3_-) and nitrite (NO_2_-) residuals on the surface of materials [72]. When paired with the results of biocompatibility testing, this analysis of surface residuals can support the establishment of acceptable limits for residuals on the surfaces of NO_2_-exposed materials. Five control samples, five Mid-Range-exposed samples and five High-Range-exposed samples were evaluated for surface residuals. Control samples, Mid-Range-exposed samples, and High-Range-exposed samples were placed in extraction bags with water and incubated at 50 °C for 72 h +/- 2 h, following ISO 10993-12 instructions for material extraction [66]. After extraction, the extraction media were placed in cold storage (2 °C to 8 °C) until testing. Uncertainty, calculated using a 95% confidence interval test, is reported.

Cytotoxicity testing was performed by Millstone Medical Testing (Bloomfield, CT, USA) using the elution method described in ANSI/AAMI/ISO 10993-5: 2009/(R) 2014 [73]. A material may be considered non-cytotoxic with a grade of 0, 1, or 2, as shown in Table 15. A control sample and a High-Range-exposed sample were tested for cytotoxicity for all six polymers, and a Mid-Range-exposed sample was tested for three of the polymers.

## 5. Conclusions

Medical devices and drug delivery devices require materials that are compatible with terminal sterilization. The current portfolio of medical device polymers represents a form of natural selection driven by sterilization requirements. Over decades, materials incompatible with EO or gamma radiation have been phased out or reformulated, leaving a universe of polymers that reliably withstand these processes. NO_2_ sterilization joined the sterilization landscape with a need to partner with materials suppliers to investigate polymers and polymer additives that are compatible with NO_2_ as well as other sterilization methods like H_2_O_2_.

These detailed results will aid device designers and regulators in the types of chemical reactions that may occur with polymers during NO_2_ sterilization. The data demonstrate the compatibility of Amorphous Polyester, PSU, Polycarbonate, PEEK, PAEK, and Polypropylene with the NO_2_ sterilization process at room temperature. Despite the oxidizing properties of NO_2_, as well as it being a free radical, reactions between NO_2_ and these polymers require overcoming activation-energy barriers, which can be achieved only at elevated temperatures [16,24]. No significant chemical changes were observed, and the mechanical testing showed no changes in their morphologies, mechanical properties, or surface hardness. Even though Amorphous Polyester had a low signal for radicals, the additional data showed no significant changes, indicating that the radicals were not a source of degradation for the material. Color changes were observed with the Polypropylene and Polycarbonate samples, which were graded as moderate. All other samples exhibited only a slight change that would be only noticeable to trained observers or under controlled conditions. The color change seen with materials may be due to reactions between NO_2_ and additives in the polymer blends. Based on our results, none of the samples tested had a residual sterilant density greater than 90 µg/cm^2^. Additionally, unlike EO-sterilized materials and H_2_O_2_-sterilized materials, none exhibited cytotoxicity, whereas more than 1 µg/cm^2^ of residual EO or H_2_O_2_ is unacceptable.

This evaluation of six materials demonstrates the rigorous testing and analytical methods needed to establish material compatibility. The materials tested for this report are compatible with use in drug delivery systems and medical devices. The methodology presented in this study can be applied to evaluate additional polymer systems for compatibility with NO_2_ sterilization. Ongoing work is evaluating additional materials to build the library of material compatibility and will include 1-year real-time aging results from the materials reported here.

## Figures and Tables

**Table 1:** Summary of Materials, Suppliers, Molecular Structures, and Their Uses in Medical Devices.

Material	Supplier	Molecular Structure/Functional Group	Used in (Medical Device)
Amorphous Polyester	Ensinger	Contains ester (–COO–) linkage; amorphous, transparent structure	Rigid trays for pre-filled syringes, packaging of devices and pharmaceutical products, implants [25,26]
Polysulfone (PSU)	Ensinger	Sulfone (–SO_2_–) group with aromatic rings	Catheters, access port implants, dialysis cartridges, surgical instrument trays [27,28]
Polycarbonate (PC)	Covestro	Carbonate (–O–(C=O)–O–) linkage between aromatic rings	Disposable auto-injectors, portable drug delivery devices, on-body drug delivery devices [29]
PolyEtherEtherKetone (PEEK)	Ensinger	Ketone (C=O) and ether (–O–) groups in aromatic backbone	Implantable drug-delivery pumps, on-body e-drug delivery devices, surgical instruments, orthopedic implants [30,31]
PolyArylEtherKetone (PAEK)	Syensqo	Aromatic backbone with ether and ketone groups	Structural components of drug-delivery devices, orthopedic surgical tools, orthopedic implants [32,33,34]
Polypropylene (PP)	Ensinger	Aliphatic hydrocarbon chain with methyl (–CH_3_) side groups	Syringes, IV bags, catheters, Intranasal delivery actuator [35,36]

**Table 2:** Average Achieved Cycle Parameters with Uncertainty Values.

Group	Vacuum Level (Torr)	NO_2_ Injection (mg/L)	Relative Humidity (%RH)	NO_2_ Exposure Time (mm:ss)	Exposure Temperature (°C)	Exposure Pressure (Torr)	No. of Pulses
Mid-Range	20	15.4 ± 0.1	79.6	15:00	24.43 ± 0.11	592	6
High-Range	20	15.3 ± 0.1	79.6	15:01	24.54 ± 0.07	592	12
Within Tolerance (Y/N)	Y	Y	Y	Y	Y	Y	Y

**Table 3:** Summary of average glass transition temperatures for each amorphous polymer sample comparing unexposed control, Mid-Range-exposed, and High-Range-exposed. Literature-reported glass transition temperature ranges are included for reference.

Polymer	Literature Reported T_g_ (°C)	Measured T_g_ Control (°C)	Measured T_g_ Mid-Range-Exposed (°C)	Measured T_g_ High-Range-Exposed (°C)
Amorphous Polyester	67–125	105.1 ± 0.1	103.7 ± 0.5	103 ± 2
Polysulfone (PSU)	180–230	191.3 ± 2.0	189.2 ± 2.5	187.4 ± 3.0
Polycarbonate (PC)	140–155	146.1 ± 0.9	146.7 ± 0.4	146.3 ± 0.7

**Table 4:** Summary of average melting transition temperatures for each semi-crystalline polymer sample comparing unexposed control, Mid-Range-exposed, and High-Range-exposed. Literature-reported melting transition temperature ranges are included for reference.

Polymer	Literature Reported T_m_ (°C)	Measured T_m_ Control (°C)	Measured T_m_ Mid-Range-Exposed (°C)	Measured T_m_ High-Range-Exposed (°C)
PolyEtherEtherKetone (PEEK)	340–350	343.0 ± 0.7	343 ± 2	343.5 ± 0.2
PolyArylEtherKetone (PAEK)	300–400	344 ± 4	341 ± 3	342 ± 6
Polypropylene (PP)	160–166	159.1 ± 0.6	159.9 ± 0.3	160 ± 3

**Table 5:** Average percent difference in crystallinity for Mid-Range-exposed and High-Range-exposed from control average percent crystallinity for semi-crystalline polymer samples.

Polymer	Mid-Range-Exposed Percent Difference in Crystallinity (%)	High-Range-Exposed Percent Difference in Crystallinity (%)
PolyEtherEtherKetone (PEEK)	−0.6 ± 2	−0.1 ± 0.6
PolyArylEtherKetone (PAEK)	−1.5 ± 5	1.6 ± 3
Polypropylene (PP)	0.4 ± 0.4	−0.35 ± 0.09

**Table 6:** Summary of average true yield strength, true strength at fracture, and true percent elongation at fracture for each polymer comparing unexposed control samples, Mid-Range-exposed samples, and High-Range-exposed samples.

Polymer	Group	Yield Strength (MPa)	Strength at Fracture (MPa)	Elongation at Fracture (%)
Amorphous Polyester	Control	49.9 ± 0.9	112 ± 4	92 ± 2
	Mid-Range-Exposed	50.7 ± 0.4	110 ± 6	90 ± 3
	High-Range-Exposed	50.1 ± 0.6	110 ± 4	90 ± 2
Polysulfone (PSU)	Control	89.8 ± 1.0	56.2 ± 3.6	14 ± 3
	Mid-Range-Exposed	90.2 ± 0.3	59.1 ± 2.0	14 ± 2
	High-Range-Exposed	88.0 ± 2.4	55.5 ± 1.8	13 ± 1
Polycarbonate (PC)	Control	65.6 ± 0.3	117 ± 12	68 ± 5
	Mid-Range-Exposed	64.9 ± 0.5	112 ± 19	65 ± 10
	High-Range-Exposed	64.7 ± 0.6	120 ± 13	69 ± 6
PolyEtherEtherKetone (PEEK)	Control	116 ± 1	81 ± 3	16 ± 2
	Mid-Range-Exposed	117 ± 1	82 ± 1	16.3 ± 0.9
	High-Range-Exposed	117.2 ± 0.3	80 ± 2	13.7 ± 0.8
PolyArylEtherKetone (PAEK)	Control	101 ± 10	94 ± 7	39 ± 10
	Mid-Range-Exposed	95 ± 5	90 ± 3	30 ± 8
	High-Range-Exposed	112 ± 6	112 ± 6	50 ± 5
Polypropylene (PP)	Control	39 ± 4	41 ± 2	37 ± 8
	Mid-Range-Exposed	37 ± 5	42 ± 2	40 ± 6
	High-Range-Exposed	35 ± 3	38 ± 3	36 ± 5

**Table 7:** Summary of average Shore D Hardness measurements for each polymer comparing control, Mid-Range-exposed, and High-Range-exposed.

Material	Control	Mid-Range-Exposed	High-Range-Exposed
Amorphous Polyester	78 ± 0	78 ± 1	79 ± 1
Polysulfone (PSU)	85 ± 0	85 ± 0	86 ± 0
Polycarbonate (PC)	85 ± 0	84 ± 1	85 ± 0
PolyEtherEtherKetone (PEEK)	89 ± 1	89 ± 1	90 ± 0
PolyArylEtherKetone (PAEK)	88 ± 0	89 ± 0	88 ± 0
Polypropylene (PP)	75 ± 0	75 ± 0	76 ± 0

**Table 8:** Summary of average color change (ΔE) for each polymer comparing control with Mid-Range-Exposed and with High-Range-Exposed.

Material	ΔE Between Control and Mid-Range-Exposed	ΔE Between Control and High-Range-Exposed
Amorphous Polyester	1.1 ± 0.3	2.0 ± 1.1
Polysulfone (PSU)	1.0 ± 0.3	0.5 ± 0.1
Polycarbonate (PC)	3.1 ± 0.1	4.1 ± 0.1
PolyEtherEtherKetone (PEEK)	1.2 ± 0.1	1.9 ± 0.2
PolyArylEtherKetone (PAEK)	0.8 ± 0.1	1.8 ± 0.1
Polypropylene (PP)	3.7 ± 0.5	4.8 ± 0.1

**Table 9:** Values of nitrate or nitrite surface residuals on each polymer across the control, Mid-Range-Exposed, and High-Range-Exposed samples.

Material	Nitrate Levels (µg/cm^2^)	Nitrite Levels (µg/cm^2^)
Amorphous Polyester	90.8 ± 3.1	0.065 ± 0.004
Polysulfone (PSU)	70.7 ± 2.7	0.029 ± 0.004
Polycarbonate (PC)	35.1 ± 0.4	<LOQ
PolyEtherEtherKetone (PEEK)	18.8 ± 0.9	0.073 ± 0.000
PolyArylEtherKetone (PAEK)	16.5 ± 0.1	0.076 ± 0.005
Polypropylene (PP)	0.412 ± 0.070	0.265 ± 0.027

**Table 10:** Cytotoxicity grades for each polymer.

Material	Control Grade	Mid-Range-Exposed Grade	High-Range-Exposed Grade
Amorphous Polyester	0	-- *	0
Polysulfone (PSU)	0	-- *	0
Polycarbonate (PC)	0	0 **	0
PEEK	0	-- *	0
PAEK	0	-- *	0
Polypropylene	0	0	1

* Mid-Range-Exposed samples were not tested for these materials since the High-Range-Exposed samples were graded 0. ** The control and High-Range-Exposed samples for Polycarbonate had to be retested, in the meantime, the Mid-Range-Exposed sample was also tested.

**Table 11:** EPR testing material information and testing results.

Test	Material	Material Supplier	Samples Tested	Signal Found
Initial	Amorphous Polyester (Tecadur MT TR)	Ensinger	1	Yes
Retest, Dry Ice Storage			1	No
Retest, Dry Ice Storage			2	1 Yes 1 No
Retest, Dry Ice Storage	Semi-Crystalline Polyethylene Terephthalate Glycol (PETG)	E&T Plastics	3	No
	Crystalline Polyester (Tecapet PET Natural)	Ensinger	3	No

**Table 12:** ΔE Thresholds for Perceptible Color Change.

ΔE Value	Perceptibility	Description
<1	Not perceptible	Color difference is imperceptible to the human eye
1–2	Slight	Only noticeable to trained observers or under controlled conditions
2–3	Small	Visible when colors are compared side-by-side
3–5	Moderate	Noticeable under normal viewing conditions
5–10	Significant	Clear color difference; easily perceived
>10	Major	Colors appear distinctly different

Note: These thresholds are based on the CIEDE2000 color difference formula, which is considered the most accurate for human visual perception [54].

**Table 13:** List of Polymeric Material Suppliers.

Material	Product Name	Supplier
Amorphous Polyester	Tecadur MT TR	Ensinger (Nufringen, Germany)
Polysulfone (PSU)	Tecason S Natural Udel P1700	Ensinger (Nufringen, Germany)
Polycarbonate (PC)	Makrolon 2458	Covestro (Leverkusen, Germany)
PolyEtherEtherKetone (PEEK)	Tecapeek Natural	Ensinger (Nufringen, Germany)
PolyArylEtherKetone (PAEK)	Avaspire AV651	Syensqo (Brussels, Belgium)
Polypropylene (PP)	Tecapro MT	Ensinger (Nufringen, Germany)

**Table 14:** Material Exposure Cycle Conditions.

Group	Vacuum Level (Torr)	NO_2_ Injection (mg/L)	Relative Humidity (%RH)	NO_2_ Exposure Time (mm:ss)	Exposure Temperature (°C)	Exposure Pressure (Torr)	No. of Pulses
Mid-Range	20	15	80	15:00	24	590	6
High-Range	20	15	80	15:00	24	590	12
Tolerance	±10	±1.5	±10%	±00:10	±1.5	±30	±0

**Table 15:** Cytotoxicity Test Scoring.

Grade	Reactivity	Conditions of all Cultures
0	None	Discrete intracytoplasmic granules, no cell lysis, no reduction in cell growth.
1	Slight	Not more than 20% of the cells are round, are loosely attached and without intracytoplasmic granules, or show changes in morphology; occasional lysed cells are present; only slight growth inhibition observable.
2	Mild	Not more than 50% of the cells are round or devoid of intracytoplasmic granules; no extensive cell lysis; not more than 50% growth inhibition observed.
3	Moderate	Not more than 70% of the cell layers contain rounded cells or are lysed; cell layers not completely destroyed, but more than 50% growth inhibition was observed.
4	Severe	Nearly complete or complete destruction of the cell layers.

## Data Availability

The original contributions presented in this study are included in the article. Further inquiries can be directed to the corresponding authors.

## Abbreviations

The following abbreviations are used in this manuscript:

DPPH	2,2-diphenyl-1-picrylhydrazyl
DSC	Differential Scanning Calorimetry
EO	Ethylene Oxide
EPR	Electron Paramagnetic Resonance
FTIR-ATR	Fourier Transform InfraRed Attenuated Total Reflectance Spectroscopy
HNO_2_	Nitrous Acid
HNO_3_	Nitric Acid
H_2_O_2_	Hydrogen Peroxide
NO_2_	Nitrogen Dioxide
PAEK	PolyArylEtherKetone
PC	Polycarbonate
PE	Polyethylene
PEEK	PolyEtherEtherKetone
PETG	Polyethylene Terephthalate Glycol
PP	Polypropylene
PSU	Polysulfone
RH	Relative Humidity
SAL	Sterility Assurance Level
